# Determinants of pesticides use among tomato farmers in the Bono and Ahafo regions of Ghana

**DOI:** 10.1038/s41598-024-55169-4

**Published:** 2024-03-06

**Authors:** Joseph Bandanaa, Augustine Bosomtwe, Alexander Danson-Anokye, Eric Adjei, Matilda Bissah, Daniel A. Kotey

**Affiliations:** 1 CSIR-Plant Genetic Resources Research Institute, P. O. Box 7, Bunso, Eastern Region Ghana; 2 Department of Agriculture, Berekum West District, P. O. Box 160, Berekum, Ghana

**Keywords:** Fungicide, Insecticide, Pesticides, Tomato production, Weedicide, Environmental impact, Ecology, Environmental economics, Environmental social sciences, Sustainability

## Abstract

Tomato production plays a crucial role in the livelihoods of farmers and agricultural households in the forest savanna transitional belt of Ghana. However, the success of tomato cultivation is hindered by the presence of insect pests and diseases, necessitating the use of agricultural inputs. This study aimed to identify the pesticides used in tomato farming, assess their World Health Organization (WHO) active ingredient hazard class, determine the precautionary behaviour associated with pesticide use by tomato farmers, and elucidate the socio-economic factors influencing pesticide usage in the Bono and Ahafo regions of Ghana. A multistage sampling procedure was employed to select 1009 respondents, who were administered a structured questionnaire. Descriptive statistics and logistic regression models were used to analyse the collected data. The results revealed that tomato farmers utilized 15 types of insecticides (e.g., lambda and chlorpyrifos ethyl based), 8 types of fungicides (e.g., mancozeb and sulphur + copper based), and 6 types of weedicides (mostly glyphosate based) on their crops. Notably, four insecticides and two fungicides types were found to be unregistered products. Lambda-cyhalothrin-based insecticides and mancozeb-based fungicides were predominantly used by the farmers. The assessed pesticides exhibited varying levels of hazard, ranging from slight to moderate. The study found that farmer training was a significant driver influencing insecticide use, while the educational level of farmers and average yield played important roles in determining fungicide use. Socio-economic factors such as being the head of the household, employing farm workers, the cultivated tomato variety, and farmer training influenced weedicide use. The type of tomato variety cultivated emerged as the primary socio-economic driver of pesticide use. The study recommended the establishment and implementation of a systematic monitoring regime for pesticide product marketing and use, with the aim of reducing the utilization of unregistered products by farmers. Implementing these measures supports sustainable tomato farming in the Bono and Ahafo regions of Ghana.

## Introduction

Tomatoes (*Solanum lycopersicum*) are globally renowned for their adaptability, nutritional richness, and culinary versatility. With escalating demands for both fresh and processed tomato products, global production has expanded significantly, aided by leading producers such as China, India, the United States, Turkey, and Egypt^1^. Tomatoes are utilized in various forms, including sauces, pastes, ketchup, canned variants, and dried products, owing to their convenience and extended shelf life^2^. In Ghana, tomato cultivation plays a vital role in enhancing food security, income, and employment opportunities. However, the sector faces challenges like pest infestations, diseases, inadequate infrastructure, and market instability^3,4^. Tomatoes are integral to Ghanaian cuisine, featuring prominently in stews, soups, and sauces, while processed tomato products witness high consumption and trade activities in local markets and urban centers^5,6^. This underscores the importance of tomatoes in both global and Ghanaian agriculture.

Tomato farming plays a vital role in the economy and food security of the Bono and Ahafo regions of Ghana, located in the middle belt of the country. The cultivation of tomatoes is particularly significant for smallholder farmers as it serves as a primary source of income and employment^7^. The forest savanna transitional belt of Ghana, including the Bono and Ahafo regions, experiences thriving tomato production^8^. Melomey et al.^5^ found that tomato farming is a major economic activity in the Bono Region, predominantly carried out by smallholder farmers. The study emphasized the efficient use of resources in tomato production, generating substantial income for farmers. Similarly, Anang et al.^9^ highlighted the importance of tomato farming for smallholders in the Ahafo Region, as well as the various constraints faced by tomato farmers which may limit their productivity and profitability.

Despite the economic and nutritional significance of tomato farming, there is a growing concern for its heavy reliance on pesticides due to vulnerability to pests and diseases is a notable challenge. Tomato cultivation in Ghana relies heavily on chemical inputs, with a focus on Dichlorodiphenyltrichloroethane (DDT), despite its ban from the registered list of pesticides. The low-yielding nature of the production, characterized by Eshun et al.^10^, leads to high dependency on chemical inputs. Farmers employ various chemicals to combat diseases, pests, and weeds, but their effectiveness is questioned, prompting the application of high dosages. This raises concerns about pesticide poisonings and fatalities in developing nations due to factors like inadequate protective measures, weak enforcement, deficient labeling, low literacy rates, and limited awareness of pesticide concentrations in vegetables^7^. The use of chemical pesticides has been noted to be ineffective, leading to the application of high dosages^10^.

Small-scale farmers rely on pesticides to protect their crops and meet market demands for blemish-free, high-quality tomato fruits. However, the excessive use of pesticides poses risks to human health and the environment while increasing production costs^11^. Furthermore, there is a lack of data on the socioeconomic factors influencing pesticide use and the hazard classification of commonly used pesticides in tomato-growing areas. Several other studies^11–13^ conducted on pesticide use by vegetable farmers in Ghana also highlighted pesticide residues, health risks, farmers' knowledge and practices, and factors influencing pesticide use decisions.

The challenges confronting tomato farmers in the Bono and Ahafo regions underscore the importance of comprehending pesticide usage determinants and advocating for sustainable farming practices. This study aims to bridge knowledge gaps by identifying pesticides in tomato farming, assessing farmers' precautionary behavior, determining the World Health Organization (WHO) hazard class for commonly used pesticide active ingredients, and exploring socioeconomic factors influencing pesticide usage among small-scale tomato farmers in these regions.

## Methodology

### Study area

**Figure 1:**
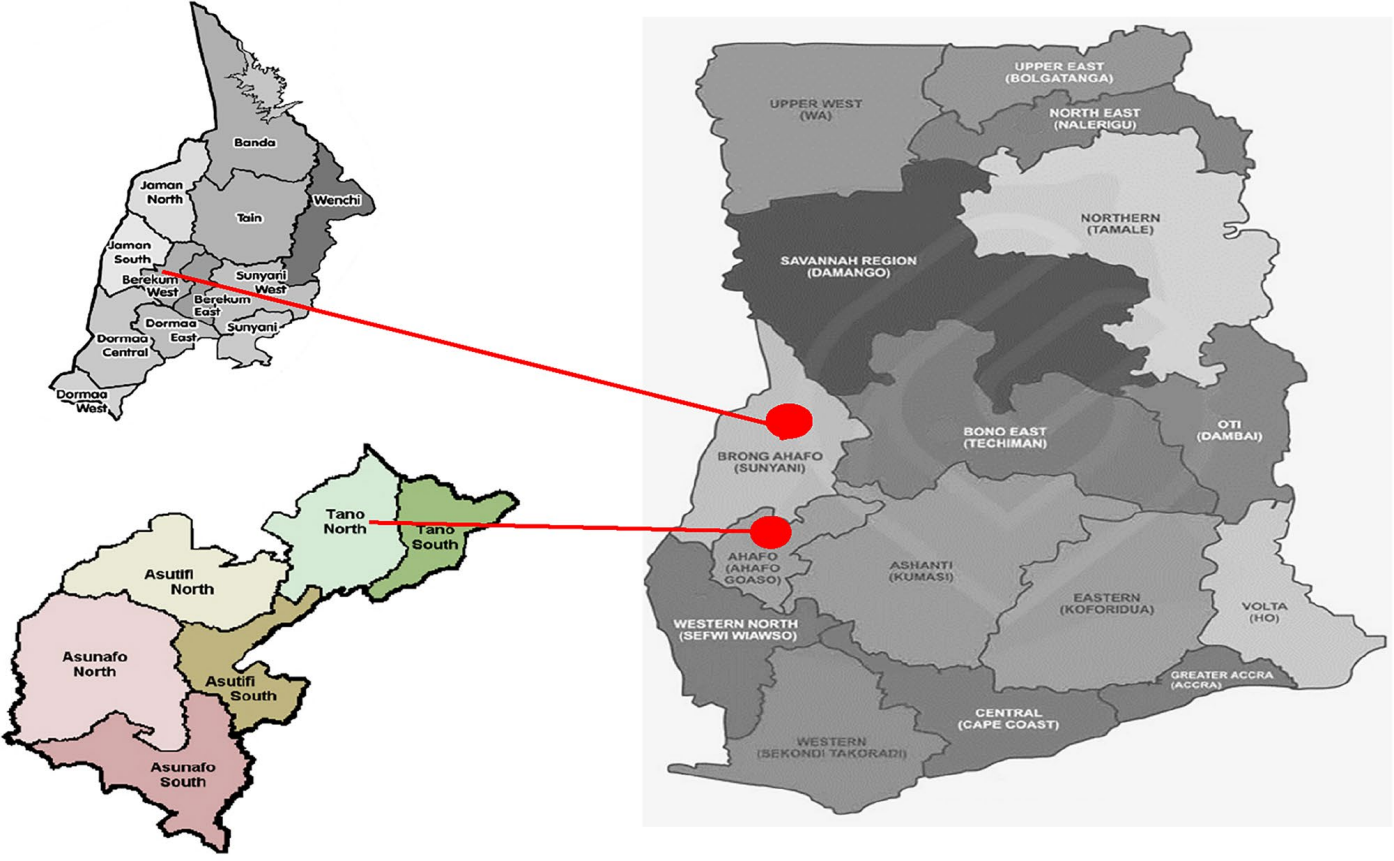
Map of the study area. Source: Ghana Local Government Services.

The study was conducted in two regions of Ghana, namely the Ahafo and Bono regions (Fig. 1).

The Ahafo Region is located within the forest belt of Ghana and is characterized by fertile soils. The Bono region, on the other hand, is known for its moist semi-deciduous forests and fertile soils. The temperature in both regions varies between 14 and 40 °C, as reported by the Ghana Statistical Service^14^. These regions were selected as the study areas due to their significance in tomato farming and their representative nature of the forest savanna transitional belt in Ghana.

### Research design and data collection

Data were gathered for this study using a cross-sectional research method. Data collection from identified individuals at a single point in time is made possible by cross-sectional design^15^. This approach was appropriate for this study since it allowed the researcher to collect data quickly and efficiently from a large group of participants with a variety of characteristics from each region. A two-stage multistage sampling technique was employed. The districts were selected based on the involvement of farmer cooperatives in tomato production. The Berekum West district was chosen in the Ahafo region, while the Tano North district was selected in the Bono region. In the first stage, the population of farmers in the study districts who practiced agriculture as a major source of livelihood was 143,072, serving as the primary sampling unit. In the second stage, the population of farmers belonging to tomato cooperatives was 142,630. Using the sampling formula in Eqs. (1) and (2), a total of 1009 was determined. One thousand and nine tomato farmers were then randomly selected.

1 $$n=\frac{N x {Z}^{2 }x p(1-p)}{\left(N-1\right)x {e}^{2 }+ {Z}^{2 } x p (1-p)}$$

where *N* = population representing only tomato farmers; Z = Z-score corresponding to 95% confidence level (1.96); p = estimated population with characteristics of interest based on prior knowledge; e = margin of error (0.03 ~ 3%); $n$ = final sample size.

Therefore,

2 $$n=\frac{\mathrm{142,630} x {1.96}^{2 }x 0.5(1-0.5)}{\left(\mathrm{142,630}-1\right)x {0.03}^{2 }+ {1.96}^{2 } x 0.5 (1-0.5)},\,\,\,\, n=1009.$$

Aside vegetables like tomato, farmers are also involved in the cultivation of cash crops like cocoa and cashew, and other annual crops including maize. The data was collected from tomato farming households using a structured questionnaire loaded electronically into tablets. The questionnaire development was based on the research objectives and literature review, and key variables to be measured. Face-to-face interviews with selected farmers were conducted by trained enumerators with support from trained agricultural field assistants.

### Data validity and reliability

The study ensures data accuracy and credibility by rigorously addressing validity and reliability concerns in questionnaire design, validation, and testing. The questionnaire was developed through literature review and expert feedback to comprehensively cover factors influencing pesticide use among tomato farmers in the Bono and Ahafo regions of Ghana. The questionnaire was pre-tested for validity and local context applicability before the data collection. Pre-testing refined questionnaire items, ensuring clarity.

### Conceptual framework

**Figure 2:**
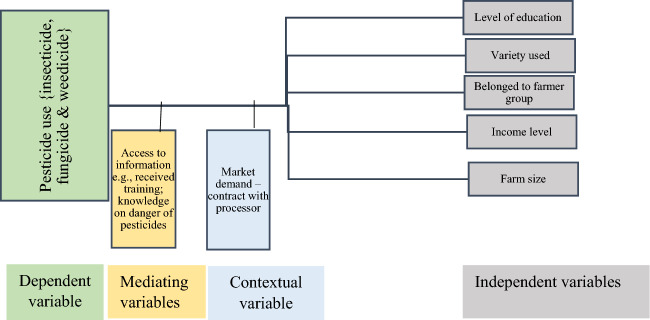
Conceptual framework.

The literature identifies gaps in understanding socio-economic factors influencing pesticide use and hazard classification in tomato-growing regions. This research aims to fill these gaps and contribute to existing knowledge. The conceptual framework (Fig. 2), rooted in theory, guides the study, delineating logical connections between variables. Independent variables like gender and education influence, while mediating variables such as training access and pesticide knowledge act as catalysts. Contextual variables, including government regulations and market demands, deepen understanding. Research questions, informed by this framework, aim to unravel complexities, and provide insights into factors governing pesticide use among small-scale tomato farmers in the Bono and Ahafo regions.

The socio-economic determinants of pesticide use by tomato farmers involves understanding the various factors that influence their decision-making process. Apart from the dependent variable (pesticide use), the other variables include the independent variables (gender, level of education etc.), mediating variables (access to training, knowledge on dangers of pesticides), and contextual variables (Government regulations and policies, and market demand—contract with processor).

### Data analysis

#### Descriptive statistics

STATA was used to generate descriptive statistics. The analysis of the data produced means and frequencies that described the households in the study sites. The frequencies and percentages were established for categorical variables including demographic characteristics, protective behaviour, and the different types of pesticides used.

### Analytical framework

The quantitative data collected was also analysed using the STATA statistical software^16^. For this study, the socio-economic determinants of insecticides, fungicides and weedicides used were analysed using the Logistic Regression model. Since the dependent variables, the use of insecticides, fungicides and weedicides are dichotomous, the logistic regression model was used. The logistic model was estimated according to Hosmer et al.^17^ in Eq. (3) and the parameters defined in Table 1.

**Table 1:** Summary of variables and parameters used estimating determinants of pesticides use.

Variable description	Measurement	A priori expectation
Dependent variables		
Insecticide use (yes/no)	1 = use; no use = 0	
Fungicide use (yes/no)	1 = use; no use = 0	
Weedicide use (yes/no)	1 = use; no use = 0	
Explanatory variables		
Region of tomato farmer	1 = Ahafo; Bono = 0	-
Sex of farmer	1 = female; male = 0	+
Head of household	1 = head; 0 = otherwise	+
Age of farmer	1 = youth; 0 = aged	-
Marital Status of farmer	1 = married; 0 = otherwise	+
Education of farmer	1 = educated; 0 = none	+
In any other occupation aside tomato farming	1 = yes; 0 = no	-
Registered member of cooperative	1 = yes; 0 = no	+
Average yield (tonnes)	1 = greater than 2 tonnes; 0 = less than 2 tonnes	+
Employ workers on farm	1 = yes; 0 = no	+
Tomatoes variety use	1 = improved variety; 0 = local variety	+
Knowledge on danger of pesticides	1 = yes; 0 = no	+
If the farmer has received training on pesticides use	1 = yes; 0 = no	+

Source: Authors construct (2023).

The implicit logistic model:

3 $${{Y}_{{\text{i}}}=\upbeta }_{0 }+{\upbeta }_{1}({\text{Region}})+{\upbeta }_{2}({\text{Gender}})+{\upbeta }_{3}({\text{HeadofHousehold}})+{\upbeta }_{4}({\text{WhichAgeGroupdoyoubelong}})+{\upbeta }_{5}({\text{MaritalStatus}})+{\upbeta }_{6}({\text{LevelofEducationcompleted}})+{\upbeta }_{7}({\text{Areyouengagedinanyotheroccupationasidetomatofarming}})+{\upbeta }_{8}({\text{Areyouaregisteredmemberofanycooperativeinthecommunitydistrict}})+{\upbeta }_{9}({\text{farmsize}})+{\upbeta }_{10}({\text{AverageYieldperkgacreforthepastthreeyears}})+{\upbeta }_{11}({\text{emplyworkers}})+{\upbeta }_{12}({\text{varietyUSe}}3{\text{Years}})+{\upbeta }_{13}({\text{dangerKnowledge}})+{\upbeta }_{14}({\text{processorContract}})+{\upbeta }_{15}({\text{Training}})+{\upbeta }_{16}({\text{income}})+{\upvarepsilon }_{{\text{i}}}$$

where Y_i_ is the dependent variable measured as a dummy, 1 if the tomato farmer has used pesticides [insecticides, fungicides, and weedicide], 0 if the tomato farmer has never used pesticides [insecticides, fungicides and weedicide]; ${\upbeta }_{0}$ is the constant term; ${\upbeta }_{1}$ to ${\upbeta }_{16}$ represent the coefficients of the explanatory variables; and ${\upvarepsilon }_{{\text{i}}}$ the error term. The a priori expectations for ${\upbeta }_{1}$ to ${\upbeta }_{16}$ are presented in Table 1. The coefficients of the explanatory variables were estimated using STATA software^16^.

## Results and discussions

The socioeconomic data on the farmers utilized for this study included information on gender, household size, marital status, level of education, ownership of land, farm size, farmer protective behaviour related to pesticide usage, and the types of pesticides farmers used (Table 2). Tomato farming is male dominated in the two regions. For most households, the members were less than 5. The respondents interviewed are mostly married. Literacy level among the respondents was high as majority have had a minimum of basic education. The land used for tomato farming is mostly rented, with sizes between 1 and 2 acres.

**Table 2:** Descriptive statistics among tomato farmers and regions.

Variable	Farming system		Regions	
	Tomatoes only	Mixed crops	Ahafo	Bono
Gender				
Male	364 (36)^1^	440 (44)	333 (33.0)	471 (46.7)
Female	102 (10)	103 (10)	76 (7.5)	129 (12.8)
Household size				
More than 14	2 (0.2)	3 (0.3)	3 (0.3)	2 (0.2)
10–14	46 (4.6)	11 (1.1)	53 (5.3)	4 (0.4)
5–9	159 (15.8)	175 (17.3)	201 (19.9)	133 (13.2)
Less than 5	259 (25.7)	354 (35.1)	152 (15.1)	461 (45.7)
Marital status				
Divorced	9 (0.9)	15 (1.5)	8 (0.8)	16 (1.6)
Married	359 (35.6)	428 (42.4)	372 (36.9)	415 (41.1)
Separated	29 (2.9)	19 (1.9)	10 (1)	38 (3.8)
Single (never married)	67 (6.6)	77 (7.6)	15 (1.5)	129 (12.8)
Widowed	2 (0.2)	4 (0.4)	4 (0.4)	2 (0.2)
Level of education				
Basic/JHS/MLSC	280 (27.8)	323 (32.0)	362 (35.9)	241 (23.9)
Degree and above	1 (0.1)	19 (1.9)	15 (1.5)	5 (0.5)
Diploma	2 (0.2)	24 (2.4)	18 (1.8)	8 (0.8)
No formal education	72 (7.1)	79 (7.8)	60 (5.9)	91 (9.0)
SHS/TVET	110 (10.9)	96 (9.5)	142 (14.1)	64 (6.3)
Tertiary	1 (0.1)	2 (0.2)	3 (0.3)	0 (0.0)
Land ownership status				
Communal land/purchased	110 (10.9)	49 (4.9)	111 (11)	48 (4.8)
Inherited	28 (2.8)	122 (12.1)	51 (5.1)	99 (9.8)
Lease hold/rent	328 (32.5)	327 (36.9)	247 (24.5)	453 (44.9)
Farm size				
0.5 acre or less	12 (1.2)	23 (2.3)	6 (0.6)	29 (2.9)
1.0–2.0 acres	239 (23.7)	228 (22.6)	209 (20.7)	258 (25.6)
2.5–3.5 acres	151 (15.0)	131 (13.0)	138 (13.7)	144 (14.3)
4.0–5.0	45 (4.5)	63 (6.2)	26 (2.6)	82 (8.1)
More than 5.0 acres	19 (1.9)	98 (9.7)	30 (3.0)	87 (8.6)
Average income				
Less than 330 GHC	5 (0.5)	55 (5.5)	60 (5.9)	0 (0.0)
331–500 GHC	39 (3.9)	20 (2)	53 (5.3)	6 (0.6)
501–750 GHC	51 (5.1)	23 (2.3)	57 (5.6)	17 (1.7)
751–950 GHC	66 (6.5)	102 (10.1)	117 (11.6)	51 (5.1)
More than 950 GHC	305 (30.2)	343 (34.0)	122 (12.1)	526 (52.1)

^1^Freq (%); Source: Field data (2021).

Tomato production is somewhat lucrative as majority of the respondents earn on average, more than GHC 950.00 per season.

### Pesticides use behaviour among groups of farmers

**Figure 3:**
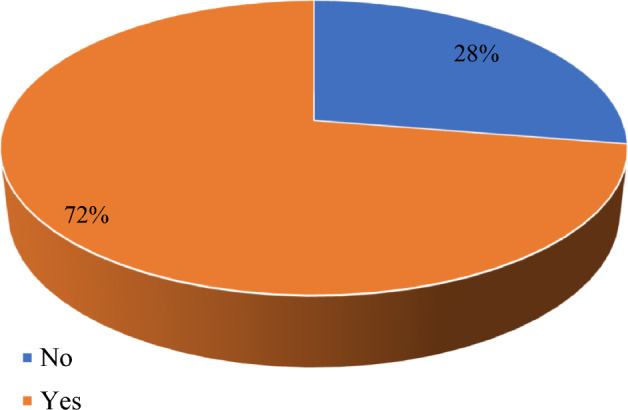
Pesticides use behaviour among tomato farmers.

In terms of knowledge on the hazards and adverse effects associated with the use of pesticides, 50.6% of the 72% of respondents who used protective cloths were aware of the dangers (Fig. 3).

The common hazards and adverse effects mentioned by respondents were related to body itching, body weakness and dizziness, and perceived cause of infertility among both men and women. This according to Segal and Giudice^18^, and Pizzorno^19^, are due to environmental toxins, which can have a profound impact on reproductive health and fertility.

### Pesticides used in tomato production in the study regions

The analysis revealed that 15 insecticides, eight (8) fungicides and six (6) weedicides were used on tomatoes by farmers in the Bono and Ahafo regions of Ghana (n = 1009) (Table 3). Out of the 15 insecticides used, four (4) were not registered^20^. Also, two (2) fungicides used by the respondent tomato farmers was not registered by the Environmental Protection Agency (EPA) of Ghana. The study analysis indicated that most of the tomato farmers interviewed used lambda-cyhalothrin based insecticides. For tomato farmers in Ahafo, emamectin benzoate-based insecticides were frequently used next to lambda-cyhalothrin. Condifor, an insecticide for controlling capsid, bugs and insect pests in cocoa pods was found in use for tomato production in some sampled areas accounting for 2.7% and 2.2% in Ahafo and Bono regions, respectively. This observation is consistent with findings of Dari et al.^7^ in Upper East and Northern, where tomato farmers used confidor (same active ingredient as condifor) for managing insect pests of tomato. Dari et al.^7^ terms the practice misapplication of insecticide because of the physiologically variance of the crops. Mancozeb based fungicides were frequently used in the two regions next to an unregistered fungicide known commonly as “datin”. Almost all the weedicides used were glyphosate based. A few respondent farmers used atrazine. Paraquat dichloride-based weedicides were also mostly used by tomato farmers in Bono region.

**Table 3:** Pesticide types used by tomato farmers in the Ahafo and Bono regions.

Pesticides type	Commercial name	Region [n (%)]		Active ingredient (AI)	WHO AI hazard class^1^
		Ahafo	Bono		
Insecticides	Aceta star	4 (0.4)	24 (2.4)	Bifenthrin + acetamiprid	II
	Attack	148 (15)	23 (2.3)	Emamectin benzoate	II
	Bomec	0 (0)	38 (3.9)	Abamectin	II
	Bypel	6 (0.6)	82 (8.3)	Perisrapae granulosis virus + Bacillus thuringiensis	II
	Condifor	27 (2.7)	22 (2.2)	Imidacloprid	II
	Ema star	0 (0)	11 (1.1)	Emamectin benzoate	II
	Golan	34 (3.4)	0 (0)	Acetamiprid	II
	K-optimal	0 (0)	23 (2.3)	Acetamiprid + lambda-cyhalothrin	II
	Lambda	161 (16.3)	146 (14.8)	Lambda-cyhalothrin	II
	Strike super	0 (0)	15 (1.5)	Chlorpyrifos and cypermethrin	II
	Sunpyrifos	7 (0.7)	118 (12)	Chlorpyrifos ethyl	II
	Lounder	0 (0)	20 (2)		
	Dietin	0 (0)	43 (4.4)		
	Topsane	10 (1)	0 (0)		
	Poultry 10	0 (0)	25 (2.5)		
Fungicides	Cuprofix	0 (0)	85 (9)	Mancozeb + metallic copper	II
	Funguran	37 (4)	4 (0.4)	Copper hydroxide	III
	Mancozeb	102 (11)	166 (18)	Mancozeb	III
	Ridomil Gold	4 (0.4)	4 (0.4)	Mefenoxam	III
	Topcop	3 (0.3)	171 (18.2)	Sulphur + copper	III
	Trimangol 80 WP	0 (0)	4 (0.4)	Maneb	III
	Sulphur 8	1 (0.1)	15 (1.6)		
	Datin	199 (21)	143 (15)		
Weedicides	Adwumawura	31 (4.1)	19 (2.5)	Glyphosate	III
	Atrazine	3 (0.4)	3 (0.4)	Atrazine	III
	Glyphosate	3 (0.4)	4 (0.5)	Glyphosate	III
	Gramoxone	53 (7)	204 (27)	Paraquat dichloride	II
	Kingkong	11 (1.5)	4 (0.5)	Glyphosate Mono ammonium salt	III
	Sunphosate	95 (12.5)	328 (43.3)	Glyphosate	II

^1^Class I = highly hazardous, II = moderately hazardous, III = slightly hazardous; source: Field data (2021).

Except for trade names that were not registered, the pesticides used by the tomato farmers in the two regions were classified under either class II or III according to the WHO chemical active ingredient hazards classification. Generally, all insecticides used were within the WHO category II classification. This implies that, apart from unregistered trade names, whose category could not be determined, insecticides used by tomato farmers posed moderate hazards to human health and the environment. Likewise, in Ethiopia, Mergia et al.^21^ discovered that pesticides classified as World Health Organization (WHO) class II (moderately toxic) were the most employed. Apart from “datin”, which was not a registered fungicide, the frequently used mancozeb based fungicide was classified in the WHO hazard class III. This implies that about 11% of tomato farmers interviewed in the Ahafo region and 18% of tomato farmers in the Bono region use fungicides that pose slight health and environmental hazards. With regards to weedicides, almost all were slightly hazardous based on the WHO active ingredient hazard classification^22^. Likewise, Tambe et al.^23^ discovered that tomato farmers in Cameroon, a West African nation, predominantly face chemical hazards and encounter health issues related to their work.

### Socio-economic determinants of pesticides use by tomato farmers

Results of the logistic regression model to identify the drivers of pesticides (insecticides, fungicides, and weedicides) use among tomato farmers are presented in Table 4. Using the specifications with the dependent variables measured as 1, if the tomato farmer has used a pesticide i.e., insecticides, fungicides, and weedicides, 0 if the tomato farmer has never used a pesticide i.e., insecticides, fungicides, and weedicides. A maximum likelihood procedure was used to estimate the parameters. The co-efficient of determination, Pseudo R^2^ of 0.2520, indicate that about 25.2% of the variation in the use of insecticides by tomato farmers could be explained by the explanatory variables in Table 4. The co-efficient of determination, Pseudo R^2^ of 0.6933, indicate that about 69.3% of variation in fungicide use by tomato farmers could be explained by the explanatory variables. The co-efficient of determination, Pseudo R^2^ of 0.4248, indicate that about 42.5% of variation in weedicide use by tomato farmers could be explained by the explanatory variables. The three models for determining the drivers of insecticide, fungicide, and weedicide use, respectively are statistically significant at 1%.

**Table 4:** Estimations of regression models for drivers of pesticides use by tomatoes farmers.

Variables	Insecticide use			Fungicide use			Weedicide use		
	Co-efficient	Standard error	Marginal effects	Co-efficient	Standard error	Marginal effects	Co-efficient	Standard error	Marginal effects
Region	–	–		1.664	1.216	0.001	0.827	0.555	0.016
Gender	− 0.408	0.815	− 0.002	− 0.713	0.645	− 0.001	− 0.058	0.379	− 0.001
Head of household	− 0.679	0.734	− 0.002	1.792**	0.647	0.001	1.751***	0.378	0.064
Age of farmer	1.261*	0.700	0.006	− 0.933	0.899	− 0.001	− 1.033**	0.410	− 0.020
Marital Status of farmer	1.085	0.662	0.006	0.418	0.727	0.001	0.735*	0.399	0.019
Education of farmer	1.441**	0.731	0.010	0.047***	0.479	0.001	0.453	0.319	0.011
In any other occupation aside tomato farming	− 1.122	0.740	− 0.005	2.030*	0.655	0.001	0.738**	0.328	0.016
Registered member of cooperative	0.455	0.647	0.002	2.568	1.555	0.002	− 0.241	0.398	− 0.005
Average yield (tonnes)	− 1.551*	0.838	− 0.006	− 0.302***	0.505	− 0.001	− 0.642**	0.327	− 0.013
Employ workers on farm	–	–	–	3.667**	1.109	0.006	1.746***	0.437	0.569
Tomatoes variety use	− 1.387**	0.686	− 0.011	− 4.043**	1.662	− 0.016	− 2.722***	0.476	− 0.189
Knowledge on danger of pesticidess	− 1.008	0.772	− 0.004	2.653**	0.997	0.002	0.197	0.331	0.004
Received training	1.901***	0.621	0.013	2.418	1.035	0.002	2.877***	0.401	0.136
Model diagnostics									
Observations	1009			1009			1009		
Pseudo R^2^	0.252			0.693			0.425		
Prob > Chi2	0.000***			0.000***			0.000****		

Standard errors in parentheses, *** p < 0.01, ** p < 0.05, * p < 0.1. Source: Field data (2021).

Farmer training was the main driver of insecticide use. Participation of farmers in training programs, an important mediating variable, exposes them to information on target pests, application doses and frequencies. Thus, farmers who have attended training programs could have relevant information on insecticides which can influence how they use them. In research conducted in Zambia by Goeb and Lupi^24^, it was found that training was associated with increased comprehension among farmers regarding pesticides and their beliefs concerning pesticide hazard management. This training also resulted in better safety practices among farmers, leading to a decrease in their exposure to pesticides while working. Likewise, Mwatawala and Yeyeye^25^ observed in Tanzania that trained farmers were more inclined to use insecticides compared to their untrained counterparts. Similarly, studies conducted beyond Africa, such as those by Zhou et al.^26^ and Damalas and Koutroubas^27^, underscore the significance of training in augmenting farmers' understanding of pesticides. In Pakistan, Khan et al.^28^ noted that farmers participating in training programs were inclined to utilize insecticides as plant protectants. The level of education of the farmer, and tomato variety cultivated were statistically significant at 5% indicating that tomato farmers were 1% more likely to use insecticides based on their educational level and 1% less unlikely to use insecticides if the farmer cultivates a local tomato variety. Education creates awareness as has been observed by Mwatawala and Yeyeye^25^. While the age of respondent farmer was positively significant at 10%, results showed average yield was negatively significant at 10%. Thus, tomato farmers were more likely to use insecticides with a decrease in their yield. Nevertheless, although not statistically significant, the marital status of the farmer, and membership of the farmer in a cooperative were positively related to insecticide use.

The educational level of the farmer and average yield (tonnes/ha) were the main drivers of fungicide use.

This is plausible especially for yield since the overriding concern among farmers is crop loss attributed to pests and diseases (including fungi). Economic losses resulting from this motivates farmers to use fungicides to safeguard their crop from loss. Zhang et al.^29^ argued that farmers who anticipated higher harvests with more fungicide use had a higher probability of overusing fungicides. Similarly, Jallow et al.^30^ observed that farmer`s perception of pesticide (including fungicides) uses as a prerequisite for attaining higher yields played a vital role as a driving factor for use in Kuwait. Headship of a household, ability to employ farm workers, tomato variety cultivated and knowledge on the dangers of pesticides were statistically significant at 5% indicating that tomato farmers who are head of their household, employed workers on their farms, were aware of the danger of pesticides and used improved tomato varieties were 1–2% more likely to use fungicides. While tomato farmers involved in other occupations aside tomato farming was positively significant at 10%, training, the region from which the farmer originated, marital status and membership of a cooperative were positively related to fungicide use. This was however not statistically significant.

Tomato farmer being head of a household, employment of farm workers, tomato variety cultivated (especially improved varieties), and receipt of training were the main drivers of weedicide use. The age of farmer, average yield (tonnes/ha) and engagement in other occupation aside tomato farming were statistically significant at 5% indicating that tomato farmers who are aged and have average yields that are less than 2 tonnes/ha, were 1–2% more likely to use weedicides. The marital status of tomato farmers was positively significant at 10%. Nevertheless, the region the farmer originated from, the educational level of the farmer and knowledge of the danger of pesticides were positively related to weedicide use but not statistically significant.

## Limitations of the study

Acknowledging potential limitations and the time gap between data collection and publication, the study on determinants of pesticide uses among tomato farmers in the Bono and Ahafo regions of Ghana remains relevant and significant in addressing pressing agricultural challenges and advancing scholarly understanding. Agricultural practices and challenges in these regions are likely persistent, highlighting the continued relevance of understanding factors influencing pesticide use among tomato farmers. The study's findings have implications for agricultural policy and extension services in Ghana, particularly in promoting safer pesticide use practices and enhancing access to alternative pest management strategies. By identifying socio-economic determinants and contextual factors influencing pesticide use behaviours, the study contributes to the existing knowledge on sustainable agriculture, pest management, and rural livelihoods in Ghana and beyond.

Despite its relevance, the study may face limitations related to self-reporting bias by farmers, who might underreport or overreport their pesticide usage or adherence to safety measures. Additionally, the study's cross-sectional design provides a snapshot of pesticide usage and associated factors at a specific point in time, limiting insights into the dynamics of pesticide usage patterns and their determinants over time compared to a longitudinal study.

## Areas for further research

Conducting longitudinal studies in the future to track changes in pesticide use practices among tomato farmers over time would offer valuable insights into agricultural decision-making dynamics and the effectiveness of interventions promoting sustainable farming practices. Additionally, supplementing quantitative data with qualitative methods, like interviews or focus group discussions, can deepen understanding of the socio-cultural factors influencing pesticide use behaviours and mitigate self-reporting bias among farmers.

## Conclusion and recommendations

The study aimed to identify the pesticides utilized in tomato farming and evaluate their hazard classification according to the World Health Organization (WHO). Additionally, it sought to examine the precautionary measures taken by tomato farmers regarding pesticide usage and investigate the socio-economic factors influencing their decisions in the Bono and Ahafo regions of Ghana.

Results revealed that most tomato farmers in the surveyed areas were conscious of the negative impacts of pesticide application and practiced precautionary measures such as wearing protective gear. A diverse array of insecticides and fungicides were observed in use, with insecticides typically posing moderate health and environmental risks, while fungicides posed slightly lower risks. Some farmers in the Ahafo and Bono regions were found to employ unregistered pesticides.

Furthermore, the study identified the choice of tomato variety as a significant socio-economic determinant influencing the use of insecticides, fungicides, and weedicides. Farmer training emerged as a key mediating factor affecting insecticide application, while the educational level of farmers and average yield per hectare played pivotal roles in fungicide use. Household leadership by the farmer and the employment of farm labourers were primary drivers of weedicide application.

In light of these findings, recommendations are made for the Plant Protection and Regulatory Services Directorate (PPRSD) of the Ministry of Food and Agriculture, in conjunction with the Ghana Environmental Protection Agency (EPA), to intensify supervision and monitoring efforts. This would help curb the utilization of unregistered and potentially hazardous pesticides, considering the significant health and environmental risks they pose. Additionally, continuous education initiatives led by Agriculture Extension Officers were advised to promote safe and judicious pesticide practices among tomato farmers. Strengthening both mediating and contextual variables was deemed essential in mitigating the adverse health outcomes associated with pesticide usage.

## Data Availability

The datasets generated during and/or analysed during the current study are available from the corresponding author on reasonable request.
